# The emerging regulatory potential of SCF^Met30 ^-mediated polyubiquitination and proteolysis of the Met4 transcriptional activator

**DOI:** 10.1186/1747-1028-3-11

**Published:** 2008-07-25

**Authors:** Srikripa Chandrasekaran, Dorota Skowyra

**Affiliations:** 1 Edward A. Doisy Department of Biochemistry and Molecular Biology, Saint Louis University School of Medicine, St. Louis, MO, 63104, USA; 2 Department of Biochemistry and Biophysics, University of North Carolina, Chapel Hill, NC, 27599, USA

## Abstract

The yeast SCF^Met30 ^ubiquitin ligase plays a critical role in cell division by regulating the Met4 transcriptional activator of genes that control the uptake and assimilation of sulfur into methionine and S-adenosyl-methionine. The initial view on how SCF^Met30 ^performs its function has been driven by the assumption that SCF^Met30 ^acts exclusively as Met4 inhibitor when high levels of methionine drive an accumulation of cysteine. We revisit this model in light of the growing evidence that SCF^Met30 ^can also activate Met4. The notion that Met4 can be inhibited or activated depending on the sulfur metabolite context is not new, but for the first time both aspects have been linked to SCF^Met30^, creating an interesting regulatory paradigm in which polyubiquitination and proteolysis of a single transcriptional activator can play different roles depending on context. We discuss the emerging molecular basis and the implications of this new regulatory phenomenon.

## Review

**Figure 1:**
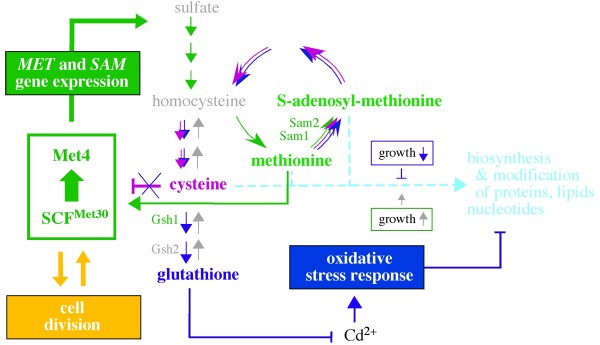
**Roadmap highlighting the relationship between the SCF^Met30^-Met4 interplay and the regulatory schemes involved in sulfur assimilation, oxidative stress and cell division**. The model emphasizes the notion that the SCF^Met30 ^ubiquitin ligase activates the Met4 transcriptional activator in a manner linked to cell division and dependent on low levels of methionine, allowing expression of the *MET *and *SAM *genes encoding the enzymes assimilating sulfur into methionine and S-adenosyl-methionine, respectively (green). In a negative feedback response driven by methionine accumulation, with the biosynthesis of S-adenosyl-methionine and cysteine necessary in this regulatory context, Met4 activation by SCF^Met30 ^is blocked, leading to Met4 inhibition (purple). Navy blue indicates steps involved in oxidative stress response induced by cadmium (Cd^2+^). Note that Sam1 and Sam2 enzymes are necessary for all three types of responses, emphasizing the special regulatory role of S-adenosyl-methionine biosynthesis. Methionine, S-adenosyl-methionine and cysteine are necessary for the biosynthesis and modification of proteins, lipids and nucleotides during growth (turquoise). See text for details.

In free-living single-celled organisms like yeast, metabolic pathways are exquisitely tuned to the availability of nutrients in the environment. The likelihood of sudden changes in availability of metabolites has led such organisms to acquire complex and dynamic feedback mechanisms that link nutrient availability to the control of biosynthetic pathways and to cell proliferation. The sulfur assimilation pathway is emerging as one of the most interesting examples of such a system, with the Met4 transcriptional activator involved in the uptake and assimilation of sulfur into the amino acids methionine and S-adenosyl-methionine (Fig. 1, green) regulated not only by the metabolites, but also by cell division and oxidative stress-mediated responses.

**Figure 2:**
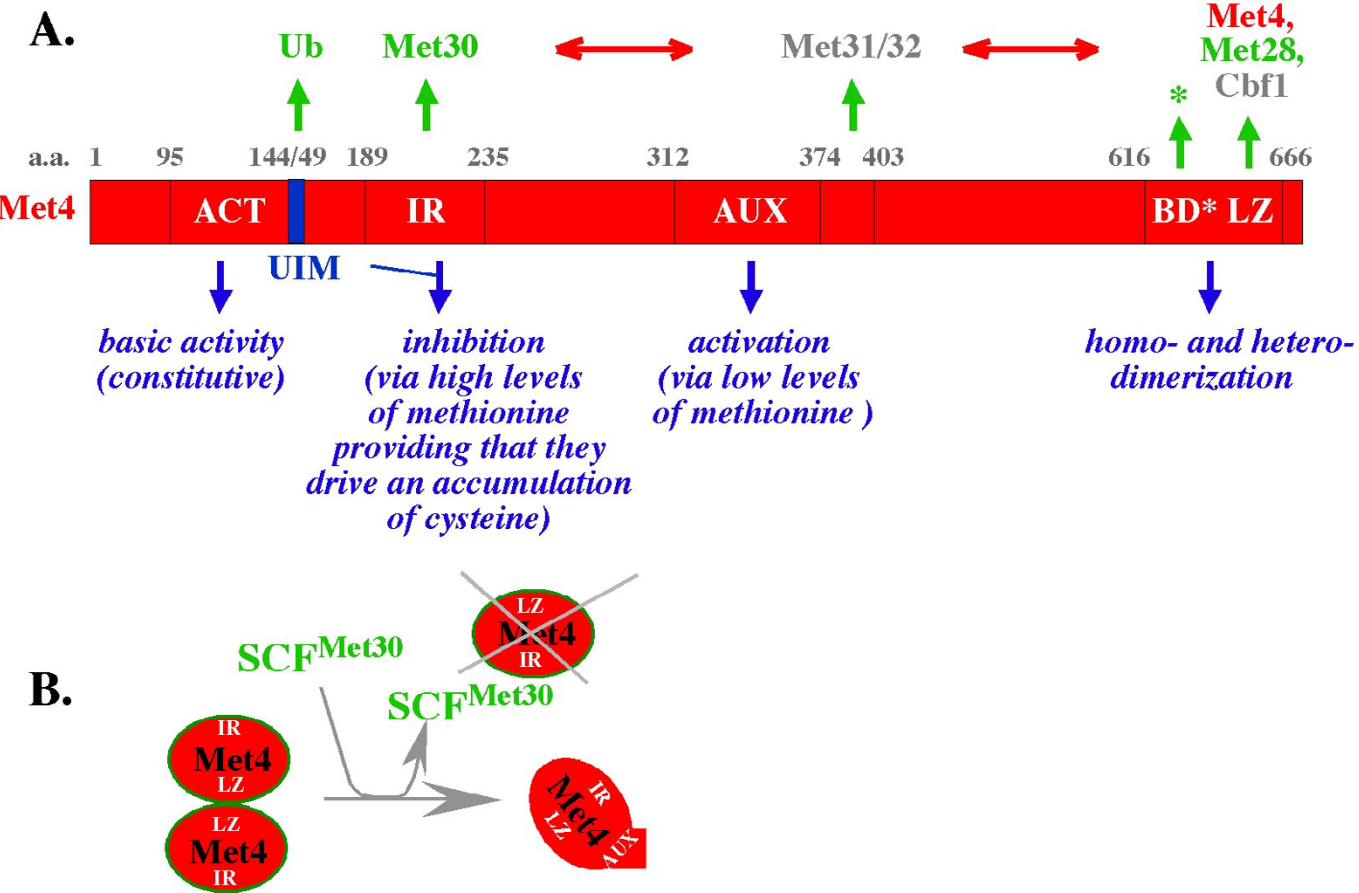
**Functional domains in the Met4 protein**. (A). Functional domains in the Met4 protein: ACT (activation domain), IR (Inhibitory Region), AUX (Auxiliary Region), BD (Basic Domain), LZ (Leucine Zipper), UIM (Ubiquitin Interacting Motif). Blue outlines functions associated with the domains. Green arrows point to cofactors interacting with the domains. Cofactors with a regulatory role are marked in green. DNA binding cofactors are marked in gray. Red horizontal arrows emphasize that new interactions could be created between cofactors when they bind to Met4. (B). Scheme illustrating dissociation of Met4 homodimers by SCF^Met30^. See text for details.

Analysis of Met4 function has first helped to establish the view that transcriptional activation is driven not only by binding of specific cofactors to activating sequences located upstream of promoters, but also by the assembly of highly specific multi-protein complexes (reviewed in [1]). Unlike most other basic leucine zipper proteins, Met4 cannot bind DNA directly due to an unusual arrangement of its basic domain (Fig. 2A, BD*), explaining why its recruitment to specific promoters depends on an interaction with the DNA binding cofactors Cbf1 and/or Met31/32 (Fig. 2A, gray). The benefits of the interaction are mutual, as Met4 regulates DNA binding by Cbf1 in a manner dependent on an interaction with the basic leucine zipper cofactor Met28 (Fig. 2A, green), which does not bind Cbf1 directly and only weakly interacts with DNA [2]. A Met28-dependent conformational transition in Met4 could thus be responsible for the stimulation of DNA binding by Cbf1. The regulatory nature of the Met4-Met28 interaction is further illustrated by the finding that more Met28 binds Met4 during growth in a methionine-free medium [3], when the Met4-controlled *MET *and *SAM *genes are expressed, correlating with the recruitment of SAGA and mediator complexes [4]. Met32 can co-purify with Cbf1/Met4/Met28 located at promoters that do not contain the specific Met32-binding element [5], suggesting that Met32, like Met28, can play a yet unidentified, DNA binding independent regulatory role.

None of these effects have initially been linked to the regulation of the Met4 activation of transcription ACT domain, which is constitutively active when fused to the LexZ DNA binding domain, but in the context of Met4 protein can be inhibited (by the inhibitory region IR) or activated (by the auxiliary AUX region) depending on the sulfur metabolite context (Fig. 2A; [6]). Instead, regulation of the ACT domain has been attributed to the Met30 F-box subunit of the ubiquitin-ligase SCF^Met30^, which via direct binding to the IR region (Fig. 2A, green) recruits Met4 for ubiquitination by the Cdc34 ubiquitin-conjugating enzyme [7-9]; reviewed in [10,11]). Consistent with its interaction with the IR region, SCF^Met30 ^has first been implicated in repression of the Met4-controlled genes during exposure to high levels of methionine, with the biosynthesis of S-adenosyl-methionine and cysteine being obligatory in this regulatory context [12]. While some investigators linked this effect to Met4 proteolysis [9,13,14,3] and others to polyubiquitination only [15-18,3], both groups placed SCF^Met30 ^in a negative feedback response aimed at lowering the levels of sulfur metabolites via lowering the expression of Met4-controlled enzymes. It is disputable whether this role explains the essential nature of the link between Met4 regulation by SCF^Met30 ^and cell division, as *MET4 *gene can be deleted without a major phenotype as long as methionine is supplied in the growth medium [15]. In contrast, the *MET30 *gene cannot be deleted without a loss of viability unless accompanied by a simultaneous deletion of the *MET4 *or *MET32 *gene [19]. The SCF^Met30^-Met4 interplay thus represents an interesting regulatory conundrum, in which some yet undefined aspect of the process of polyubiquitination and/or proteolysis rather than the removal or inhibition of the regulated protein *per se *plays an essential regulatory role.

A possible clue to the essential aspect of Met4 regulation by SCF^Met30 ^is suggested by the recent observations that SCF^Met30 ^function can be regulated by [3] and regulate [this work] the interaction between Met4 and cofactors, including Met32 (Fig. 2A, red arrows), and that SCF^Met30 ^can either inhibit or activate Met4 depending on the sulfur metabolite context [3]. The emerging mechanism underlining these effects suggests a model in which Met4 activation is the primary aspect of its regulation by SCF^Met30 ^(Fig. 1, green), while the inhibitory effect of cysteine results from a block in the activation mechanism (Fig. 1, purple). Before discussing this model, the molecular basis on which it is based, and its implications, we first discuss the results of recent quantitative proteome and metabolite profiling analyses [20], which provide an interesting insight into the main regulatory schemes involved in the control of sulfur metabolism.

### Quantitative proteome and metabolite profiling analyses clarify the role of transcriptional regulation in sulfur assimilation

At the heart of the negative feedback response that controls Met4 function is the expectation that repression of the Met4-controlled *MET *(Fig. 1, sulfate -> methionine) and *SAM *(Fig. 1, methionine -> S-adenosyl-methionine) genes in response to an accumulation of methionine lowers the levels of sulfur metabolites both in the sulfate/methionine and in the cysteine/glutathione part of the pathway. Contrary to this expectation, recent quantitative proteome and metabolite profiling analyses by Lafaye and colleagues [20] reveal that the levels of the methionine-derived sulfur metabolites (Fig. 1, methionine-> S-adenosyl-methionine -> cysteine -> glutathione) do not drop under conditions inducing repression of the *MET *and *SAM *promoters.

The way this surprising finding has been explained is that the high concentration of methionine necessary for Met4 inhibition is sufficient to drive nearly normal sulfur assimilation even when the levels of Met4-controlled enzymes are compromised. This possibility is likely, because expression of the Sam1 and Sam2 enzymes that are necessary for the conversion of methionine into S-adenosyl-methionine, while reduced under the conditions of Met4 inhibition, is still considerable (Fig. 1, methionine -> S-adenosyl-methionine, purple arrow) and the levels of sulfur metabolites are typically well below the K_m _values of the respective enzymes [20]. This explanation also agrees with the observation that the *MET4 *gene can be deleted without a major phenotype when methionine is supplied in the growth medium [15], indicating that methionine is converted into cysteine and glutathione in the absence of Met4 function.

These observations have three general implications for how sulfur assimilation is regulated. First, if the biosynthetic enzymes are not saturated, changing the pools of precursors and products would be sufficient to drive rapid metabolite fluxes. The accumulation of cysteine in response to an uptake of extra-cellular methionine (Fig. 1, purple) and the variations in sulfur metabolite pools under different culture conditions could both be explained by this mechanism [20]. Second, by uncoupling sulfur assimilation from transcriptional regulation the repression of Met4-controlled genes would make sulfur assimilation even more susceptible to regulation via the pools of precursors and products. Among its benefits, this type of regulation would be directly linked to changes in the rate with which the metabolites are utilized during growth (Fig. 1, turquoise) and could be buffered by the levels of glutathione, the end product of sulfur assimilation (Fig. 1, navy blue). Indeed, glutathione can accumulate more than any other sulfur metabolite (2–15 mM vs <4 μM homocysteine, 30–80 μM for most sulfur metabolites and 500 μM methionine), allowing sulfur accumulation when it is available in the environment and serving as sulfur reserve during starvation [21,20].

Conversely, if the enzymes are not saturated, activation of Met4 controlled gene expression could modify the *status quo *established by the pools of precursors and products. An example of such an effect is the activation of Met4-controlled *MET *and *SAM *promoters during the entry into exponential growth observed in yeast cultured in the presence and absence of methionine [3]. The way this observation could be explained is that upon growth activation, more methionine and cysteine is redirected to protein biosynthesis (Fig. 1, turquoise, growth up), modifying the *status quo *established at the previous growth rate. Another example is the yeast Cd^2+ ^response driven by the necessity to produce large amounts of glutathione that chelates the metal ions and exports them to vacuole for detoxification [22]. This response overcomes the inhibitory effect of high cysteine levels and reduces the synthesis of sulfur-rich proteins (Fig. 1, navy blue) concurrently inducing expression of Met4-controlled genes (Fig. 1, green; [23,24,20]). As a result, sulfur metabolism aimed at the production of methionine and cysteine for protein synthesis (Fig. 1, turquoise) is redirected to the synthesis of glutathione (Fig. 1, navy blue).

In summary, according to the ever-changing demands of growth and stress responses, transcriptional regulation of the Met4-controlled genes either increases the sensitivity to (via repression), or modifies the outcome of (via activation) the regulation by rapid sulfur metabolite fluxes. While the accumulation of cysteine driven by high levels of methionine controls the regulatory aspect associated with transcriptional repression, cell growth and/or stress responses regulate sulfur metabolism primarily via activation of the Met4-controlled promoters.

### Challenging the view that SCF^Met30 ^plays only a negative role in the regulation of Met4

The initial interpretation of analyses regarding the role of SCF^Met30 ^in Met4 regulation was driven by the assumption that SCF^Met30 ^acts exclusively as a negative regulator during an accumulation of cysteine. However, recent evidence suggests that SCF^Met30 ^can also activate Met4 and that the activation represents the primary aspect of Met4 regulation by SCF^Met30^. In this model, the effect of cysteine is viewed as a block in Met4 activation (Fig. 1, purple) that is normally dependent on methionine (Fig. 1, green), supporting the notion that SCF^Met30 ^can either inhibit or activate Met4 depending on the sulfur metabolite context [3].

The first implication for the model that transcriptional repression is not the only function of Met4 regulation by SCF^Met30 ^was the observation that SCF^Met30 ^binds Met4 under repressive and non-repressive conditions [9,3]. Turnover of Met4 protein has been next implicated in the activation of Met4-controlled promoters upon the entry into exponential growth in a medium with or without methionine [3]. The notion that cell division plays a positive role in the activation of Met4 via proteolysis (Fig. 1, orange up) agrees with the positive role of Met4 proteolysis in cell division (Fig. 1, orange down), which has long been suggested by the observation that *met30Δ *yeast arrest at the G1-S phase while simultaneous deletion of the *MET4 *gene bypasses the arrest [19]. The mechanism by which proteolysis could activate the function of a transcriptional activator is unknown, but this phenomenon appears to have a broad significance, as turnover of several transcriptional activators, including Gcn4, Gal4, and Ino2/4, has been implicated in their activity [25], and the activation domain of many transcriptional factors overlaps with the domain required for their proteolysis [26].

In light of the possibility that Met4 is activated via proteolysis, how legitimate is the view that Met4 is 'fully' active under conditions preventing its regulation by SCF^Met30^? Due to the lethal phenotype of *met30Δ *yeast [19], this view is based only on the observation that the *in vivo *active Met4^K163R ^mutant does not accumulate as polyubiquitinated protein and cannot be inhibited by SCF^Met30 ^during growth on a methionine-rich synthetic medium [16]. Ironically, the same report shows that Met4^K163R ^protein still binds SCF^Met30 ^and is highly unstable in a manner dependent on Cdc34 and SCF^Met30 ^function. Rapid turnover in response to polyubiquitination of a lysine distinct from K163 could best explain this phenomenon, suggesting that the K163R substitution prevents the inhibition, but not the activation of Met4 by SCF^Met30^.

If proteolysis is necessary for Met4 activation in the context of cell division, could it also be necessary for Met4 activation during Cd^2+ ^response, which is associated with strong up-regulation of Met4-controlled genes, including *MET30 *and *MET32 *[24]? The accumulation of Met30 protein in Cd^2+ ^treated cells indeed suggests an increased demand for SCF^Met30 ^function. On the other hand, under the conditions of Cd^2+ ^response, most of Met30 is detectable as a SCF^Met30^-free protein [27,28]. This effect could reflect SCF^Met30 ^inactivation, as it has been proposed, explaining how the Cd^2+ ^response overcomes the inhibitory effect of high cysteine levels (Fig. 1, purple with a navy blue cross). In this view, SCF^Met30 ^would not be able to participate in either inactivation or activation of the Met4-controlled genes. A different possibility is suggested by the more recent observation that Met4 proteolysis leads to the ATP hydrolysis-dependent disassembly of the proteasome and of SCF^Met30 ^(Babbitt and Skowyra, unpublished), an effect first linked to proteolysis of Sic1, the prototype substrate of the SCF^Cdc4 ^ubiquitin ligase [29]. In this view, disassembly of SCF^Met30 ^could reflect its vigorous involvement in Met4 activation via proteolysis (Fig. 1, green), linking Cd^2+ ^response to Met4 activation by SCF^Met30^.

The notion that Met4 function depends on activity of SCF^Met30 ^rather then on its inactivation is additionally supported by the finding that the *GSH1 *promoter can be activated by Cdc34 overproduction, explaining why Cdc34 has been identified as a high copy suppressor of the defect in glutathione biosynthesis associated with the *gsh2 *mutant ([30]; Fig. 1, Gsh1). Overproduction of the Cdc34 E2 necessary for SCF^Met30 ^function could not activate the *GSH1 *gene expression if the expression were dependent on SCF^Met30 ^inactivation.

### The molecular mechanism of Met4 regulation may depend on "two-stepping" with SCF^Met30 ^due to its role in Met4 monomerization

While the cofactor-free Met4 protein can be polyubiquitinated and degraded *in vitro *[3] and *in vivo *[14], the essential role of SCF^Met30 ^is most likely associated with the regulation of Met4 function in the context of transcriptional complexes.

The observation that the cofactors Cbf1, Met28, and one of the related Met31 and Met32 proteins can stabilize the interaction between Met4 and SCF^Met30 ^[3] suggested first that SCF^Met30 ^could regulate protein-protein interactions within the transcriptional complexes. This possibility is further supported by the observation that SCF^Met30 ^binding is sufficient to dissociate Met4 homodimers (Chandrasekaran and Skowyra, unpublished). During this process, one Met4 monomer remains bound to SCF^Met30^, while the other is released as a free protein (Fig. 2B). Dissociation of Met4 homodimers to free monomers can be observed in yeast extracts lacking Cbf1, Met28 and Met31 or Met32, while the monomers readily associate into large complexes when the cofactors are present [3]. The monomers do not re-dimerize spontaneously in the absence of the cofactors, suggesting that a major, not easily reversible change in Met4 structure accompanies its monomerization by SCF^Met30^. That a major structural change accompanies Met4 activation has been independently proposed based on genetic analysis of the constitutively active *Met4-1 *and *Met4-2 *mutants, which display a dominant negative phenotype in the presence of wild type Met4 [31].

**Figure 3:**
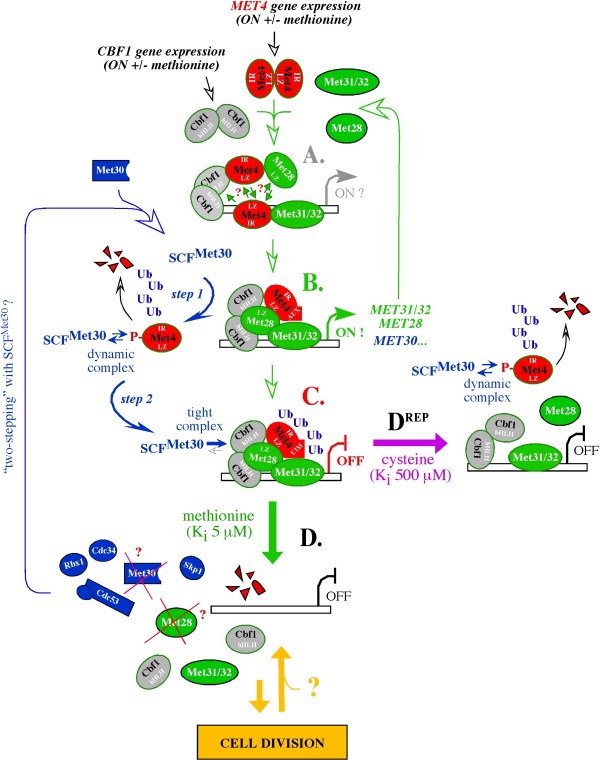
**The emerging molecular mechanism of Met4 regulation by SCF^Met30^**. The mechanism of Met4 activation by SCF^Met30 ^is based on the prediction that the formation of Met4 homodimers antagonizes proper assembly between Met4 and cofactors (A), and that the dissociation of Met4 homodimers by SCF^Met30 ^(B, *step 1*) is necessary to stabilize and/or rearrange the protein-protein interactions within the Met4/Met28/Cbf1 complex, triggering its proper assembly (B). The low abundance of SCF^Met30 ^suggests that proteolysis of the remaining Met4 molecule occupying SCF^Met30 ^(B, *step 1*) is necessary for SCF^Met30 ^recycling (B, *step 2*). As a result, 'two stepping' with SCF^Met30" ^could be necessary for each round of Met4 activity at a promoter (all steps involving SCF^Met30 ^are marked in blue). The stabilizing effect of cofactors on the SCF^Met30^-Met4 interaction ('tight complex', note exposure of the UIM domain in Met4) allows Met4 inhibition by polyubiquitination only (C), unless methionine (D) or cysteine (D^REP^) is available to destabilize the tight interaction between Met4 and SCF^Met30^, allowing Met4 proteolysis. Disassembly of the Met4 complex by the proteasome could link activation of methionine biosynthesis to cell division by releasing the DNA binding cofactors Cbf1 and Met31/32 from promoters (D), which, at least in the case of Cbf1, would make it available for its cell division role. Dissociation of Met4 prior to its proteolysis could protect the cofactors and SCF^Met30 ^from effects of the proteasome-mediated disassembly (D^REP^), preventing Cbf1 and/or Met31/32 release. See text for details.

These observations have two implications for the mechanism by which SCF^Met30 ^regulates Met4. First, the dissociation of Met4 homodimers by SCF^Met30 ^could be necessary to stabilize and/or rearrange the protein-protein interactions within the Met4/Met28/Cbf1 complex and thereby trigger its full functional potential (Fig. 3B), explaining the positive regulatory role of SCF^Met30^. Indeed, Met4 dimerization represents an interesting conundrum, as it depends on the LZ domain also implicated in binding to the basic leucine zipper (bZIP) regulatory protein Met28 and the basic helix-loop-helix (bHLH) DNA binding protein Cbf1 (Fig. 2A, LZ). As such, Met4 dimerization could antagonize proper assembly and function of the Met4/Cbf1/Met28 complex.

Second, if monomers represent an activated form of Met4 that cannot spontaneously form homodimers, degradation of Met4 monomers could be necessary to alleviate the positive effect of SCF^Met30^. As a result, 'two-stepping' with SCF^Met30 ^might be necessary for each round of Met4 activity at a promoter, with the first step facilitating Met4 activation (Fig. 3B, *step 1; *and 3B) and the second step removing the activated Met4 (Fig. 3C–D, *step 2*). In this view, turnover of Met4 protein would be an integral part of its function, with only a fraction of the total cellular Met4 degraded at a time, balancing the constitutive expression of the *MET4 *gene (Fig. 3A; [9,31]).

Finally, while degradation of the cofactor-free Met4 molecule (Fig. 3B, *step 1*) would have no immediate consequence for Met4 function at the promoter (Fig. 3B), its proteolysis could be necessary to recycle SCF^Met30^. The observation that over-expression of Met30 is sufficient to enhance repression of the Met4 controlled genes is the best illustration that SCF^Met30 ^activity is limiting *in vivo *[32]. On the other hand, over-expression of Met30 protein induces cell cycle arrest, demonstrating that low levels of Met30 are a necessary compromise associated with some other aspect of SCF^Met30 ^function. How does the abundance of Met30, which restricts the abundance of SCF^Met30^, compare with the abundance of Met4? Direct measurements of protein expression in yeast under prototrophic growth conditions suggest that Met30 is about 6-fold less abundant than Met4 (217 vs. 1300 molecules per cell, respectively; [33]). Its levels are expected to drop even more during auxotrophic growth as a result of *MET30 *gene repression and Met30 protein instability [9,32]. The significance of the fluctuations of Met30 protein and *MET30 *mRNA levels [9] still needs to be determined, but low levels of SCF^Met30 ^appear to be an intrinsic aspect of Met4 regulation. Recycling of SCF^Met30 ^molecules could thus have evolved as a mechanism controlling the regulatory potential of Met30 without the necessity to accumulate SCF^Met30^.

While the proposed role of SCF^Met30 ^in activating Met4 via dissociation of Met4 homodimers remains to be verified, this scheme illustrates particularly well how Met4 proteolysis could regulate sulfur assimilation even if the Met4 molecules at an active promoter are not polyubiquitinated or degraded, and even if the steady-state levels of Met4 do not change.

### How and why polyubiquitinated Met4 is stabilized in a proteolysis-resistant form?

Like in the case of all other known SCF substrates, polyubiquitination of Met4 has been linked to rapid proteolysis [6,9,13,14,3]. However, Met4 degradation is unusual as it can be blocked *in vivo *[15-17,13,3] and *in vitro *[3], leading to an accumulation of Met4 protein modified with the type of polyubiquitin chain that normally serves as a proteolytic signal.

Two models have been suggested for the stabilization of polyubiquitinated Met4. In a model proposed by Kaiser's group, a ubiquitin-interacting motif (UIM) motif in Met4 sequesters the polyubiquitin chain, shielding it from interaction with the proteasome [17,18]. However, Met4 can be a substrate for proteolysis despite carrying an intact UIM motif *in vivo *[6,9,14,3] and *in vitro *[3]. The UIM motif alone is thus insufficient to block degradation of polyubiquitinated Met4. In a second model, polyubiquitinated Met4 is stabilized by a mechanism dependent on assembly between Met4 and the cofactors Cbf1, Met28, and Met31 or Met32 [3]. In an unprecedented manner, the cofactors lock Met4 and SCF^Met30 ^into a tight complex active in polyubiquitination but incapable of binding the 26S proteasome (Fig. 3C, "tight complex").

The possibility that assembly between Met4, cofactors and SCF^Met30 ^controls function of the UIM domain could easily unite the models (Fig. 3C; exposure of UIM). Both models also agree that Met4 phosphorylation is necessary for its recruitment to SCF^Met30 ^[3,17,18] and that polyubiquitination of residue K163 is sufficient to inhibit Met4 activity (Fig. 3C, OFF). It is the involvement of proteolysis and its role in Met4 regulation that distinguishes the models. In the Kaiser et al. [17,18] model, the inhibition of Met4 by proteolysis-resistant polyubiquitination is the only meaningful aspect of SCF^Met30 ^function. The Chandrasekaran et al. [3] model points out that the stabilization of polyubiquitinated Met4 only delays proteolysis, keeping it in a "*stand-by" *mode for further regulation, and that depending on the sulfur metabolite context, the proteolysis can play different roles. As we discuss below, this mechanism appears to be key for defining whether Met4 protein turnover is part of its normal activity cycle (Fig. 3A–D), or reinforces the inhibitory scheme (Fig. 3D^REP^).

### Differentiating the role of Met4 proteolysis by the sulfur metabolite context

The central premise of the model proposed by Chandrasekaran et al. [3] is that stabilization and destabilization of binding between Met4, cofactors and SCF^Met30 ^is key to modulating the fate of polyubiquitinated Met4. Stabilization could trigger Met4 inhibition by polyubiquitination (Fig. 3C) unless destabilization of the complex would promote degradation of polyubiquitinated Met4 (Fig. 3D and 3D^REP^). The notion that Met4 proteolysis can play different roles is suggested by the finding that either free methionine (Fig. 3D) or cysteine (Fig. 3D^REP^) can promote degradation of polyubiquitinated Met4 by destabilizing its interaction with cofactors and SCF^Met30^, but yet the amino acids act in a different concentration range *in vitro *(5 and 500 μM, respectively) and would signal different situations *in vivo*.

The methionine-mediated proteolysis would play its role best in the standard Met4 activity cycle (Fig. 3A–D; Fig. 1, green and orange). The low K_i _for methionine (5 μM) in triggering the proteolysis of polyubiquitinated Met4 *in vitro *suggests that either low or high methionine concentration would destabilize Met4 *in vivo*. In agreement with this prediction, a high concentration of methionine is insufficient for repression of the *MET *and *SAM *promoters *in vivo *- the biosynthesis of cysteine, with the intermediate production of S-adenosyl-methionine are necessary [12]. Met4 protein is thus active at low and high methionine concentrations as long as cysteine does not accumulate, meaning that Met4 activation, while dependent on methionine, is not limited to low methionine levels. In contrast, the high K_i _for cysteine (500 μM) established in the *in vitro *studies suggests that the cysteine-mediated mechanism would engage in Met4 regulation only when cysteine accumulates above its normal (~80 μM) level, in agreement with its role in transcriptional repression [12,14]. While *in vivo *both schemes depend on biosynthesis of S-adenosyl-methionine (Fig. 1, methionine -> S-adenosyl-methionine, green and purple arrows), a highly unstable methionine metabolite, S-adenosyl-methionine does not trigger Met4 proteolysis *in vitro *[3], suggesting either that its role is indirect, or that it controls a yet undefined aspect of Met4 regulation.

How could the roles of cysteine and methionine be different if they both function via destabilizing Met4 complexes? An answer to this question may come from determining whether the same or different cofactor(s) serves as a sensor for methionine and cysteine, and whether methionine and cysteine are equally potent in dissociating cofactors from Met4. A different potency of methionine and cysteine in dissociating Met4 from cofactors could play a major role in light of the observation that proteolysis of polyubiquitinated Met4 (Babbitt and Skowyra, unpublished), like proteolysis of polyubiquitinated Sic1 [29], is associated with the robust, ATP-hydrolysis-dependent disassembly of the 26S proteasome and the Met4-interacting proteins, including SCF^Met30^. If methionine only destabilizes the interaction between Met4 and cofactors, disassembly of the transcriptional complex via the proteasome-dependent mechanism could be part of the Met4 activation cycle (Fig. 3D). In contrast, cysteine-mediated dissociation prior to Met4 proteolysis would protect the cofactors from effects of the proteasome-mediated disassembly (Fig. 3D^REP^). One major difference between these mechanisms is that only in the case of the proteasome-mediated disassembly could promoters be cleared of the DNA binding cofactors Met31/32 and Cbf1, which bind DNA even in the absence of Met4.

### What is the basis of the link between Met4 regulation by SCF^Met30 ^and cell division?

The finding that deletion of the *MET30 *gene leads to cell cycle arrest unless accompanied by deletion of the *MET4 *or *MET32 *gene, but yet the *MET4 *gene alone can be deleted without a major phenotype as long as methionine is supplied in the growth medium [19] creates an interesting regulatory conundrum. It suggests that some yet undefined, Met32-linked aspect of the SCF^Met30^-Met4 interplay rather than the inactivation and/or degradation of the Met4 protein *per se *plays an essential role. Its link to Met32 [19] has recently been verified by analysis of the Met32^Δ145-192 ^dominant suppressor of the *met30Δ *cell cycle defect, for the first time implicating Met32 in stabilization of the Met4-Cbf1 complex [5]. However, no mechanistic insight regarding the role of Met32 in the link between Met4 regulation by SCF^Met30 ^and cell division has been revealed. Similarly, analysis of the *met30ts *yeast showed that SCF^Met30 ^plays multiple roles in the cell cycle [28], but did not reveal the mechanism responsible for the cell cycle roles.

A potential mechanism by which the process of Met4 proteolysis rather then Met4 removal *per se *could be linked to cell division is suggested by the possibility that during normal Met4 activity cycle (Fig. 3A–D) the proteasome disassembles the transcriptional complexes and clears promoters from the DNA binding cofactors Cbf1 and/or Met31 or Met32 (Fig. 3D). Cbf1 (centromere binding factor 1) is of special interest as it is a dual-role cofactor necessary not only for Met4-controlled transcription [34,2,35] but also for assembly of the kinetochore [36]. If Cbf1, or other factors required for cell division, were trapped at *MET *promoters via either direct binding to DNA or via protein-protein interactions (Fig. 3B, C), they could be prevented from function in the cell division-related context until the tight interaction is destabilized. Consequently, release of such cofactors from promoters as a result of the proteasome-mediated disassembly of the Met4 complexes could link the activation of methionine biosynthesis to cell division.

In support of this model, Met4 stabilizes the Cbf1-DNA interaction in a manner dependent on Met28 [2] and Met32 [5], making its destabilization dependent either on presence of Met30, which by promoting Met4 polyubiquitination would recruit the proteasome, or on absence of *MET4 *gene, which by eliminating Met4 would prevent the Cbf1-DNA stabilization by Met28 and/or Met32. The model also agrees with the observation that expression of the Met32^Δ145-192 ^suppressor of the *met30Δ *cell cycle arrest prevents recruitment of Met4 to promoters [5], preventing Met4-dependent stabilization of Cbf1-DNA, and, possibly, Met31-DNA binding.

In principle, this simple mechanism could explain the link between Met4 turnover and cell division in the case of any cofactor that has to be shared between different cellular functions. Whether Cbf1 "mobilization" from Met4-controlled promoters indeed explains the essential nature of the SCF^Met30^-Met4 regulatory interplay in cell division and whether similar paradigm applies to the regulation of other transcriptional activators, will need to be determined.

## Conclusion

Among the emerging variety of regulatory schemes involving ubiquitination and proteolysis in transcriptional regulation [37-40] the SCF^Met30^-Met4 interplay represents one of the most interesting and complicated examples. Analyses aimed at understanding why and how SCF^Met30 ^regulates Met4 suggested first that polyubiquitination and proteolysis of a single transcriptional activator can play different roles depending on context. While recruitment of SCF^Met30 ^determines the timing of Met4 polyubiquitination, Met4 proteolysis can be delayed by stabilization of the SCF^Met30^-Met4 interaction by Met4-interacting cofactors, unless specific sulfur metabolites are available to destabilize the tight complexes. As we propose here, SCF^Met30 ^could "two-step" with Met4, first promoting its activation by dissociating Met4 homo-dimers and next removing the activated Met4. While proteolysis of the cofactor free-Met4 would have no immediate consequence for activity of the Met4 molecule located at a promoter, its degradation could be necessary to recycle SCF^Met30 ^- a concept not frequently discussed in the literature. The SCF^Met30^-Met4 regulatory interplay is also the only known example of a mechanism in which some yet undefined aspect of the process of proteolysis rather then the removal of the regulated protein *per se *plays an essential regulatory role. The emerging complexity with which the seemingly simple principle of regulated proteolysis evolved into multiple regulatory layers fits particularly well to the regulation of metabolic systems, in which timely adjustments to a variety of metabolic, stress and growth signals are necessary for survival. It will thus be not surprising if the paradigms first observed in Met4 regulation by SCF^Met30 ^played a role in other such systems.

## List of abbreviations

SCF^Met30: ^Skp1, Cdc53/Cullin, F-box protein Met30; Ub: Ubiquitin; BD: Binding domain; ACT: Activation domain; AUX: Auxiliary region; IR: Inhibitory region; Cbf1: Centromere binding factor 1; LZ: Leucine zipper; bZIP: Basic leucine zipper; bHLH; Basic helix-loop-helix.

## Competing interests

The authors declare that they have no competing interests.

## Authors' contributions

S.C. has generated some of the unpublished data reported in this manuscript, contributed to the development of the ideas and models presented here, and helped in the editorial process. D.S. is primarily responsible for conception of the presented models and ideas, for the intellectual content of the review, and for writing and editing the manuscript. Both authors read and approved the manuscript.
